# Restoration of the Oral Microbiota After Surgery for Head and Neck Squamous Cell Carcinoma Is Associated With Patient Outcomes

**DOI:** 10.3389/fonc.2021.737843

**Published:** 2021-10-06

**Authors:** Jason Y. K. Chan, Cherrie W. K. Ng, Linlin Lan, Sherwood Fung, Jing-Woei Li, Liuyang Cai, Pu Lei, Qianqian Mou, Katie Meehan, Eric H. L. Lau, Zenon Yeung, K. C. Allen Chan, Eddy W. Y. Wong, Paul K. S. Chan, Zigui Chen

**Affiliations:** ^1^ Department of Otorhinolaryngology, Head and Neck Surgery, The Chinese University of Hong Kong, Hong Kong, Hong Kong, SAR, China; ^2^ Li Ka Shing Institute of Health Sciences, The Chinese University of Hong Kong, Hong Kong, Hong Kong, SAR, China; ^3^ Department of Chemical Pathology, The Chinese University of Hong Kong, Hong Kong, Hong Kong, SAR, China; ^4^ State Key Laboratory of Translational Oncology, Sir Y.K. Pao Centre for Cancer, The Chinese University of Hong Kong, Hong Kong, Hong Kong, SAR, China; ^5^ Department of Microbiology, The Chinese University of Hong Kong, Hong Kong, Hong Kong, SAR, China; ^6^ Centre for Gut Microbiota Research, Faculty of Medicine, The Chinese University of Hong Kong, Hong Kong, Hong Kong, SAR, China

**Keywords:** microbiome, oral rinse, survival, recurrence, oral microbiome, head and neck cancer, head and neck squamous cell carcinoma

## Abstract

**Objective:**

To evaluate the dynamics of the oral microbiome and associated patient outcomes following treatment of head and neck squamous cell carcinoma (HNSCC).

**Materials and Methods:**

This was a prospective cohort study at a tertiary academic center in Hong Kong SAR of patients with head and neck squamous cell carcinoma evaluating the oral microbiome in pre- and postsurgery oral rinses (at 1, 3, and 6 months) with 16S rRNA gene V3–V4 amplicon sequencing.

**Results:**

In total, 76 HNSCC patients were evaluated. There was a significantly depressed alpha diversities of oral microbial communities observed in HNSCC oral rinse samples within the first 6 months post-surgery when compared to presurgery or healthy controls. Distant clustering between pre- and postsurgery was also observed (*p* < 0.022). Following treatment, eight oral bacterial genera showed a trend towards the restoration in the relative abundances that approximate healthy persons. In evaluating patient outcomes, the decreased relative abundance of three periodontal bacteria (*Capnocytophaga*, *Prevotella 7*, and *Leptotrichia*) and the increased relative abundance of two commensal bacteria (*Streptococcus* and *Rothia*) at 6 months postsurgery compared to presurgery showed a better 3-year disease-specific survival (a cutoff of Kaplan–Meier survival curve test *p* < 0.3 at 36 months). In particular, the postsurgery restoration of *Prevotella 7* was statistically significant in the surveyed patients (survival rate of 84% vs. 56% at 36 months, *p* = 0.0065).

**Conclusions:**

Oral microbiome dysbiosis associated with HNSCC is dynamic. These dynamics of the oral microbiome postsurgery are also associated with patient treatment and outcomes and may serve as potential biomarkers for patient management in HNSCC.

## Introduction

Disease surveillance is a challenging yet important aspect of care in head and neck squamous cell carcinoma (HNSCC). In oral cavity SCC (OSCC), approximately 20% of the patients will show recurrence, with the majority of these cases doing so within the first 2 years (1). However, the goal of surveillance is early detection, and yet it has been reported that 39% of recurrences are asymptomatic, thereby delaying diagnosis and potentially jeopardizing the possibility of salvage therapy with curative intent (1). Given the poor salvage rates of 35%, it is imperative that early detection methods of surveillance other than reliance on regular clinical examination and patients’ symptomatology, both insensitive signs of recurrence, are developed (2).

Liquid biopsies have been shown to potentially be useful in the surveillance of HNSCC (3). In nasopharyngeal carcinoma (NPC), Epstein–Barr virus (EBV) DNA in plasma has been shown to be useful in the diagnosis, prognosis, and surveillance of NPC (4–6). Similarly, human papillomavirus (HPV) DNA in plasma or oral rinses have been shown to be useful in the diagnosis and surveillance of HPV-related oropharyngeal carcinomas (7, 8). Somatic mutations of tumor DNA detected in saliva or plasma have also been shown to be useful in the detection of HNSCC and potentially useful in disease surveillance (8).

In the head and neck region, the oral bacterial microbiome has been implicated in the development of HNSCC. Poor oral health conditions including chronic periodontitis and gingivitis may result in a change in the oral microbiome leading to chronic inflammation and cancer progression (9–11). Recently, there have been studies reporting alterations in the microbiome of HNSCC (12–15). A significant loss in microbial diversity, and changes in the relative abundance of some oral bacteria, such as *Fusobacteria* and *Streptococcus*, have been observed in HNSCC patients. Our previous study has also shown that the oral microbiome may be useful in discriminating HNSCC from normal subjects by using a panel of 12 bacteria including *Fusobacterium*, *Peptostreptococcus*, *Streptococcus*, *Neisseria*, *Rothia*, *Actinomyces*, *Granulicatella*, *Oribacterium*, *Lautropia*, *Corynebacterium*, *Abiotrophia*, and *Cardiobacterium* (16). In addition, the change in the relative abundance of *F. nucleatum* was shown to be associated with patient outcomes.

Here, we sought to characterize the oral microbiome in serial oral rinses from HNSCC patients before and after surgery, with the intent of understanding the patterns of changes following HNSCC treatment. We also sought to understand the association of these oral microbiome dynamics with patient outcomes.

## Materials and Methods

### Ethics Approval

This study was approved by The Joint Chinese University of Hong Kong—New Territories East Cluster Clinical Research Ethics Committee (CREC Ref No. 2015.396, 2017.143). Patients with HNSCC who were admitted into the Prince of Wales Hospital and United Christian Hospital in Hong Kong and agreed with written informed consent were recruited between October 2015 and April 2018. All cases were reviewed by a pathologist. Separately, age-, gender-, and smoking-matched healthy individuals above 18 years of age with no history of malignancies were recruited from Prince of Wales Hospital.

### Collection of Oral Rinse Sample and DNA Extraction

Oral rinse samples of HNSCC patients were collected prior to surgery (presurgery) or after surgery (postsurgery) at 1, 3, and 6 months using 30 ml sterilized saline solution and immediately stored in −80°C, as previously described (16). Oral rinse samples from healthy subjects were collected using the same protocol. Upon DNA extraction, 1–2 ml of oral rinse solution was centrifuged at 1,600*g* for 10 min in 4°C, and the cellular pellet was further extracted for total DNA using the QIAamp DNA Mini Kit (Qiagen, USA) following the manufacturer’s protocol.

### HPV Genotyping

HPV genotyping was performed using two PCR-based amplicon sequencing assays targeting the conserved L1 open reading frame (ORF) of HPV as previously described (17). Short reads generated by Illumina MiSeq were blastn searched against a PV reference database using UPARSE software (18). An operational taxonomic unit (OTU) count table was created using a 90% identity threshold assigning each OTU with a PV type.

### 16S rRNA Gene V3–V4 Amplicon Sequencing

A minor modified primer set targeting the bacterial 16S rRNA gene hypervariable V3–V4 region (341F, 5′-CCT ACG GGN GGC WGC AG-3′; 806R, 5′-GGA CTA CNV GGG TWT CTA AT-3′) was used to PCR amplify a broad spectrum of human microbiota (19). In brief, a pair of dual unique 12-bp barcodes was indexed to each amplicon set through the forward and reverse primers modified from the Earth Microbiome Project protocol (https://earthmicrobiome.org); successful amplicons were equally pooled and sequenced on an Illumina MiSeq using paired-end 300 bp reads. Approximately 20% of PhiX control was preloaded to balance the base composition. For quality control, each sequencing batch included a mock community, DNA negative controls, and technical replicate samples.

### Microbiota Bioinformatics and Statistical Analysis

Demultiplexed short 16S reads passing the quality filter were imported into the QIIME2 package (v2019.7) to generate an amplicon sequence variant (ASV) table as previously described (19, 20). The SILVA v132 99% 16S rRNA gene reference database was used to assign bacterial identities to ASVs at phylum and genus levels. All reads assigned to archaea, mitochondria, and chloroplasts were excluded.

In order to retain all samples for diversity analysis, reads from each sample were rarefied to a depth of 5,000 mean reads, after repeating them 100 times, to normalize the data for differences in sequencing depth among samples. Bacterial genera with ≥1% relative abundance in at least one sample were retained. The diversity of observed bacterial genus taking into consideration species richness, and the effective numbers of Shannon and Simpson indexes, was calculated. Pairwise Bray–Curtis dissimilarities between samples were calculated using R v3.4.0 package. Differences in community composition were assessed using permutational multivariate analysis of variance (PERMANOVA) in the Vegan R package. Principal coordinate analysis was performed to visualize associations between community composition. Comparisons of the relative abundances of bacterial genera between defined groups were performed using nonparametric Mann–Whitney Wilcoxon rank-sum test (MWU), Wilcoxon signed-rank test (WSR), Kruskal–Wallis test (KW), or Tukey’s honest significant difference (Tukey HSD) *post-hoc* test where appropriate. Receiver operating characteristic (ROC) curve analyses, including calculation of area under the ROC curve (AUC), were used to evaluate the ability of bacterial genera to distinguish two compared groups. A Jonckheere–Terpstra (JT) trend analysis using the SAGx R package was performed to compare the consistent change in the relative abundance of oral bacteria from presurgery to each collection of postsurgery after 1, 3, and 6 months. The Kaplan–Meier method was used for univariate survival analysis, and the log-rank test was used to compare the difference in survival curves. The Cox proportional hazard regression method in a stepwise manner controlling for gender, age, postsurgery treatment, and other factors significant on univariate analysis was used for multivariate analysis of survival. A two-sided *p*-value of ≤0.05 and/or a false discovery rate (FDR)-adjusted *p*-value (q-value) of ≤0.05 was used as the threshold for significance.

## Results

### Sample and Clinical Background

Seventy-six HNSCC patients, including 45 OSCC and 31 non-OSCC, were recruited in this study. These patients provided a complete set of oral rinse samples right before surgery (presurgery, Pre) and after surgery (postsurgery) oral rinse samples at 1 (post-M1), 3 (post-M3), and 6 (post-M6) months. Meanwhile, 76 oral rinse samples from healthy controls, with matched age and gender, were recruited as a control group (Ctrl) (**Supplementary Table S1**).

### Oral Microbiota Communities Between Pre- and Postsurgery

**Figure 1:**
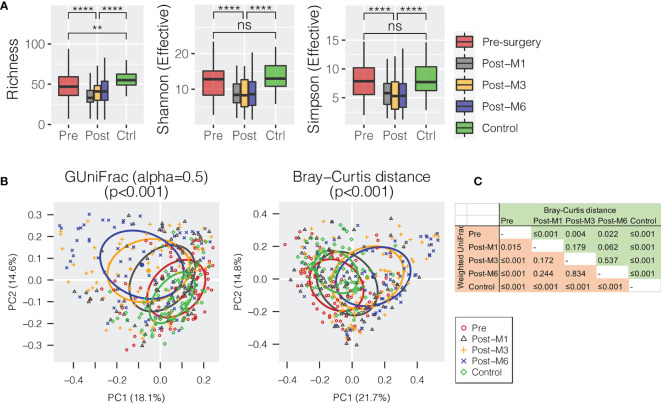
Oral microbiota diversity of HNSCC patients before surgery (presurgery) and after surgery at 1 (post-M1), 3 (post-M3), and 6 months (post-M6) compared with healthy control (Control). **(A)** Comparison of alpha diversity of oral microbiota summarized at the genus level. **(B)** Principal coordinates plot using weighted GUniFrac or Bray–Curtis dissimilarity. **(C)** Pairwise comparison of permutational multivariate analysis of variance (PERMANOVA) between different groups. The p-value is marked with ** ≤0.01, **** ≤0.0001. “ns” means no statistically significant.

All oral rinse samples were characterized for oral microbial composition using 16S rRNA V3–V4 hypervariable amplification next-generation sequencing. A total of 8,113,379 high-quality reads were generated, ranging between 5,057 and 70,019 reads per sample (21,351 ± 12,980). Overall, *Firmicutes* (relative abundance of 31.4 ± 1.0%), *Proteobacteria* (29.8 ± 1.1%), and *Bacteroidetes* (22.2 ± 0.7%) were the three most predominant bacterial phyla in the surveyed oral rinse samples, followed by *Fusobacteria* (8.9 ± 0.4%) and *Actinobacteria* (5.4 ± 0.4%) (**Supplementary Figure S1**). When bacterial taxa were summarized at the genus level (**Supplementary Figure S2**), we found significantly depressed alpha diversities of oral microbial communities in HNSCC postsurgery oral rinse samples when compared to presurgery or healthy control (**Figure 1A**). Distant clustering between pre- and postsurgery was also observed, using either the weighted GUniFrac or Bray–Curtis distances (*p* < 0.022) (**Figures 1B, C**). However, no significant difference in alpha or beta diversity was observed between the postsurgery oral microbiota collected at the different timepoints (M1 vs. M3 vs. M6).

**Figure 2:**
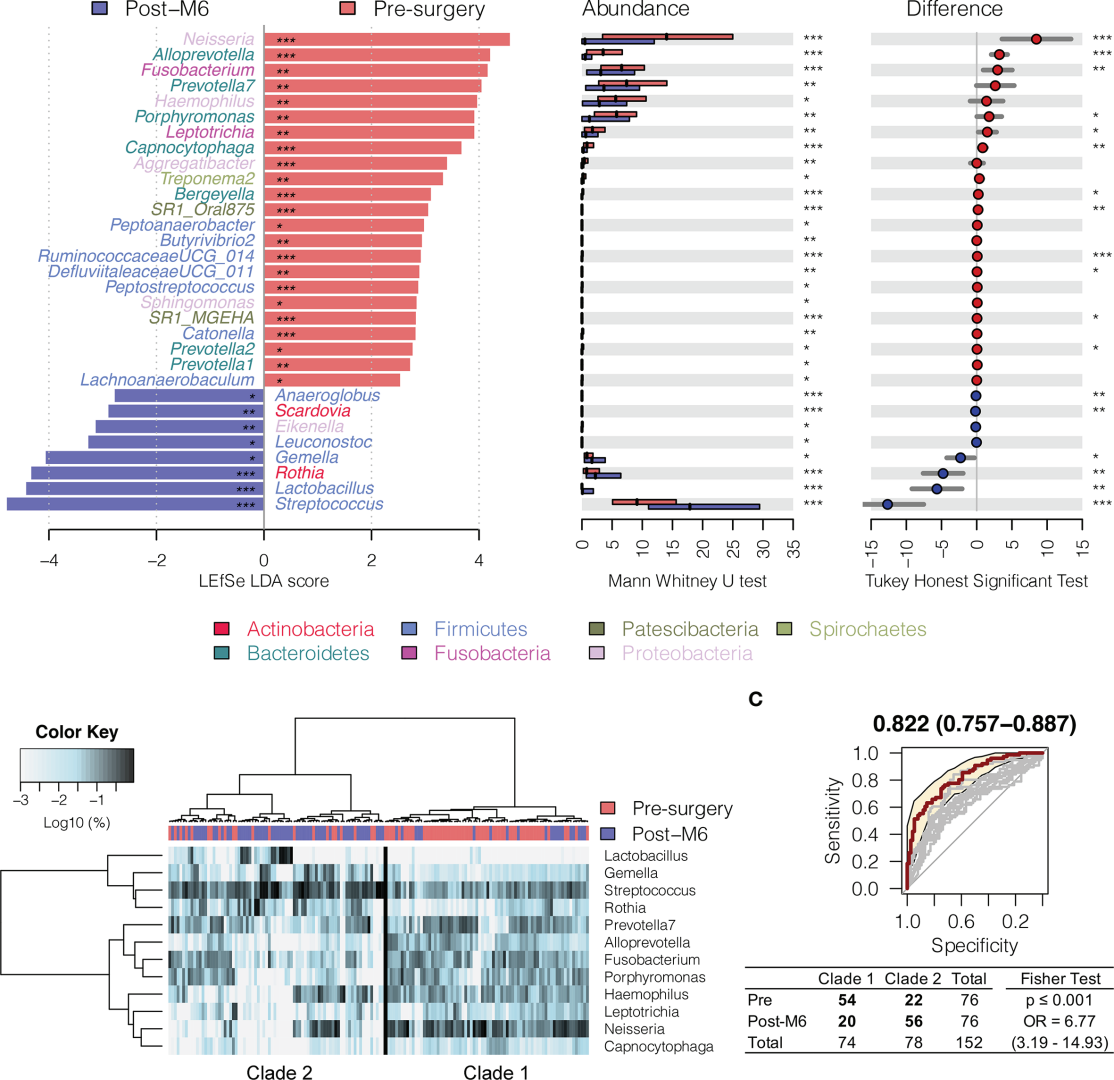
Changes in oral microbiota between HNSCC patients before surgery (presurgery) and after surgery after 6 months (post-M6). **(A)** A linear discriminant analysis (LDA) for effect size (LEfSe) of discriminative oral bacterial genera between pre- and post-M6 oral rinse samples. The bar length represents the log10 LDA score. Difference in the relative abundance tested by pairwise Tukey HSD *post-hoc* is shown on the right panel. Adjusted *p*-value is marked with * if ≤ 0.05, ** ≤ 0.01, or *** ≤ 0.001. **(B)** Hierarchical cluster analyses of oral rinses using distance matrix of 12 discriminative bacterial genera classified oral microbial communities into two clades based on the dendrogram topologies. **(C)** The receiver operating characteristic (ROC) analysis of the combined 12 genera achieved AUC of 0.822 (95 CI 0.757–0.887). Clustering between the two clades was further evaluated using a two-tailed Fisher’s exact test.

In order to compare the bacterial changes longitudinally over time, we first compared the difference in bacterial communities between oral rinse samples right before surgery and at 6 months postsurgery (**Figure 2A**). A linear discriminant analysis effect size (LEfSe) test found that the relative abundance of 23 bacterial genera was significantly depressed in HNSCC patients 6 months postsurgery (post-M6), while eight other bacterial genera were enriched [Mann–Whitney U-test *p* ≤ 0.05 and linear discriminant analysis (LDA) effect size test *p* ≤ 0.05] (**Figure 2A** and **Supplementary Table S2**). Among the bacterial genera that changed in abundance between these two time points, 12 (mean relative abundance ≥1%) were able to assign the surveyed samples into two clades, one composed of the majority of presurgery (54/74) and another the majority of post-M6 (56/78), with odds ratio of 6.77 (*p* ≤ 0.001) (**Figure 2B**). A combination of these 12 bacterial genera achieved an AUC of 0.822 (95% CI, 0.76–0.89) (**Figure 2C**).

**Figure 3:**
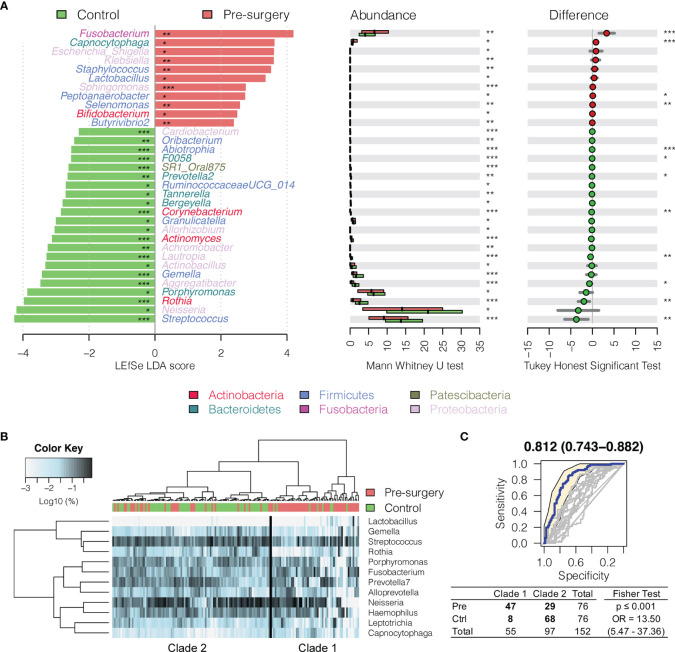
Changes in oral microbiota between HNSCC patient (before surgery) and healthy control. **(A)** A linear discriminant analysis (LDA) for effect size (LEfSe) of discriminative oral bacterial genera between HNSCC and healthy control oral rinse samples. The bar length represents log10 LDA score. Difference in the relative abundance tested by pairwise Tukey HSD *post-hoc* is shown on the right panel. Adjusted *p*-value is marked with * if ≤0.05, ** ≤0.01, or *** ≤0.001. **(B)** Hierarchical cluster analyses of oral rinses using distance matrix of 12 discriminative bacterial genera classified oral microbial communities into two clades based on the dendrogram topologies. **(C)** The receiver operating characteristic (ROC) analysis of combined 12 genera achieved AUC of 0.812 (95% CI, 0.743–0.882). Clustering between the two clades was further evaluated using a two-tailed Fisher’s exact test.

Interestingly, *Fusobacterium*, *Capnocytophaga*, *Peptoanaerobacter*, *Sphingomonas*, and *Butyrivibrio 2* were significantly decreased in relative abundance in both the 6 months postsurgery and healthy control oral rinses when compared to presurgery, while *Streptococcus*, *Rothia*, and *Gemella* were increased (**Figures 2** and **3**), suggesting partial restoration of these bacteria in relative abundance following HNSCC treatment.

### Progressive Changes in Oral Microbiota Postsurgery

**Figure 4:**
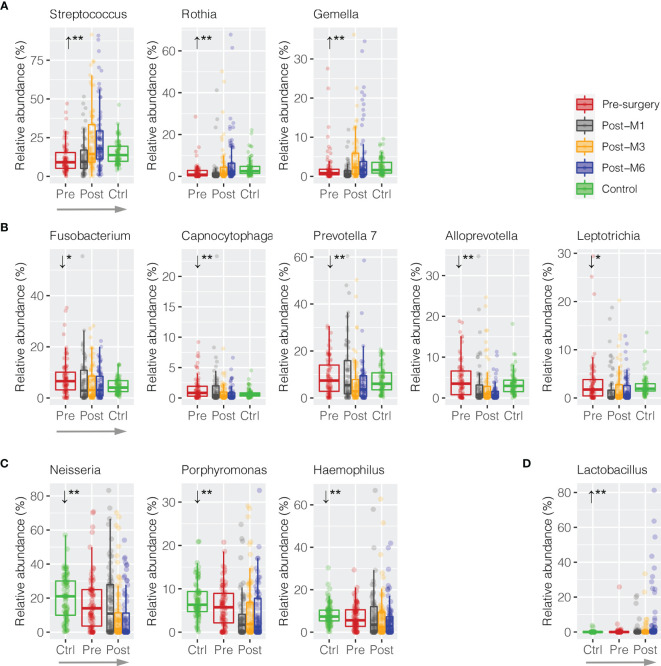
Trend analysis of discriminative oral bacteria following HNSCC postsurgery treatment. Progressive **(A)** increase or **(B)** decrease in the relative abundance of discriminative oral bacterial genera along the sequence of presurgery (pre-), postsurgery at 1 (post-M1), 3 (post-M3), and 6 months (post-M6), and healthy control (Ctrl) (presurgery → postsurgery → healthy control). Progressive **(C)** increase or **(D)** decrease in the relative abundance of discriminative oral bacterial genera along the sequence of healthy control (Ctrl), presurgery (pre-), and postsurgery at 1 (post-M1), 3 (post-M3), and 6 months (post-M6) (healthy control → presurgery → postsurgery). The p-value is marked with * if ≤0.05, ** ≤0.01.

In order to measure whether these discriminative bacterial genera had progressive changes in relative abundance along the sequence of postsurgery treatment at different time points, a trend analysis using Jonckheere–Terpstra test (JT test) was performed to compare the difference from presurgery to each collection timepoint postsurgery after 1, 3, and 6 months (presurgery → post-M1 → post-M3 → post-M6). Only bacterial genera with mean abundance ≥1% were included. As shown in **Figure 4A**, three commensal bacterial genera enriched in healthy oral rinses were also significantly enriched upon postsurgery when compared to HNSCC presurgery, including *Streptococcus* (11.44% → 12.59% → 23.26% → 24.08%, JT test *p* = 0.002), *Rothia* (2.15% → 1.98% → 4.53% → 6.91%, *p* = 0.002), and *Gemella* (2.24% → 1.23% → 4.34% → 4.53%, *p* = 0.004) (**Supplementary Table S2**). Similarly, five periodontal bacterial genera, including *Fusobacterium* (8.05% → 6.38% → 5.82% → 5.06%, *p* = 0.012), *Capnocytophaga* (1.55% → 1.49% → 0.78% → 0.69%, *p* = 0.002), *Prevotella 7* (9.01% → 10.72% → 6.55% → 6.37%, *p* = 0.004), *Alloprevotella* (4.58% → 2.98% → 2.93% → 1.36%, *p* = 0.002), and *Leptotrichia* (3.24% → 1.94% → 2.19% → 1.72%, *p* = 0.020), were depressed, although the latter three taxa had no statistical difference in relative abundance between HNSCC presurgery and healthy control (**Figure 4B**). These eight bacterial taxa represented a trend towards the restoration in the relative abundances that approximate healthy persons (presurgery → postsurgery → healthy control).

We also observed reversed progression along the sequence of healthy control → presurgery → postsurgery (**Figures 4C, D** **)**. For example, *Neisseria* was significantly depressed in HNSCC oral rinse when compared to healthy control (20.57% → 17.31%, MWU *p* = 0.025), while its abundance was further reduced after surgery at 1, 3, and 6 months (17.11% → 10.44% → 8.81%, *p* = 0.002). Similarly, *Porphyromonas* (7.46% → 6.02% → 3.45% → 4.98% → 4.23%, *p* = 0.008) and *Haemophilus* (8.46% → 7.20% → 7.99% → 6.71% → 5.79%, *p* = 0.008) were progressively decreased, while *Lactobacillus* (0.10% → 0.51% → 1.01% → 1.26% → 6.15%, *p* = 0.002) increased.

It is worth noting that three bacterial genera (*Veillonella*, *Campylobacter*, and *Pseudomonas*) had no significant changes in relative abundance before and after surgery or between HNSCC and healthy control (**Supplementary Table S2**).

### Postsurgery Clinical Features in Changing Oral Microbiota Community

**Figure 5:**
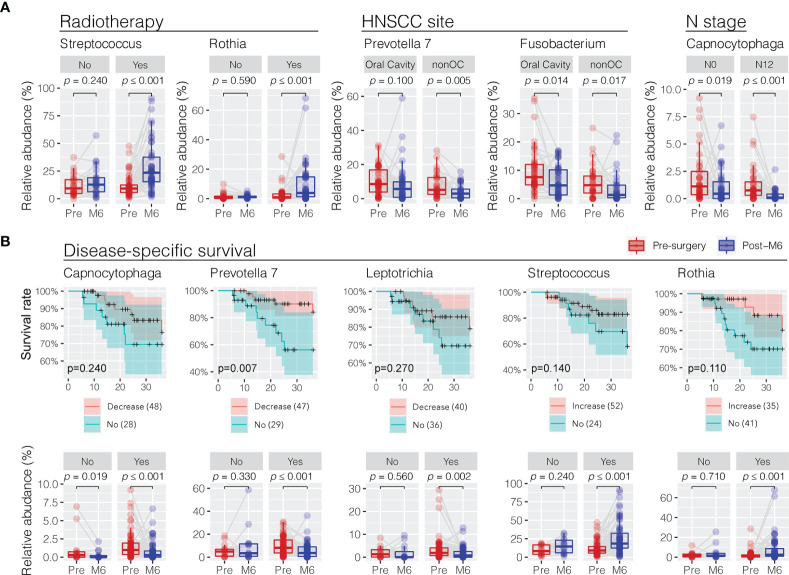
Association of trend changes in discriminative oral bacterial genera with clinical features and 3-year disease-specific survival (DSS) following postsurgery treatment. **(A)** Comparison of the relative abundance of oral bacteria related to radiotherapy (no vs. yes), HNSCC site (oral cavity vs. nonoral cavity), and N stage (N0 vs. N1/N2) before (presurgery) and after surgery at 6 months (post-M6). Univariant and multivariant regression analysis were performed to determine clinical features associated with the changes in oral microbiota in a trend towards the restoration at 6 months postsurgery, as shown in **Supplementary Table S3**. **(B)** Decrease or increase in the relative abundance of oral bacterial genera of HNSCC patients after surgery was correlated with improved 3-year disease-specific survival (DSS). A cutoff of Kaplan–Meier survival curve test *p* < 0.3 at 36 months found five bacterial genera showing significant changes in relative abundance in HNSCC patients before (pre-) and after surgery at months 6 (post-M6) with disease-specific survival. The *p*-values were calculated using Wilcoxon signed-rank test (paired Mann–Whitney Wilcoxon rank-sum test).

In order to understand the role of clinical features in influencing the changes in oral microbiota towards the postsurgery restoration, the difference in relative abundance between presurgery and post-M6 justified for confounder variables was compared using a multiple linear regression approach (**Supplementary Table S3**). Interestingly, patients who received postsurgery radiotherapy treatment had the commensal *Streptococcus* (*glm p* = 0.032) and *Rothia* (*p* = 0.035) retuning to an increased level towards the restoration similar as the healthy controls (**Figure 5A**). Similarly, the depression in the relative abundance of the periodontal *Prevotella 7* at 6 months postsurgery was more significant in non-oral cavity patients (*p* = 0.020). Although HNSCC site (oral cavity vs. non-oral cavity) and N stage (N1 and N2 vs. N0) could be the confounder factors influencing the restoration in the relative abundance of *Fusobacterium* (*p* = 0.016) and *Capnocytophaga* (*p* = 0.042) at 6 months postsurgery, respectively, we found that the decreases were significant under both variable conditions. No smoking, a higher T stage, and HPV infection might have limited confounder roles in changing the trends of oral microbiota restoration in the surveyed patients if using univariant regression analysis (**Supplementary Table S3**).

### Restoration of Oral Microbiota Associated With Disease-Specific Survival

We observed a positive association between the postsurgery restoration of oral bacteria and disease-specific survival (DSS) in the surveyed HNSCC patients. We divided the surveyed HNSCC patients into postsurgery change (increase or decrease) or no-change groups based on the abundance difference between presurgery and post-M6 relevant to one quota of the mean change of eight bacterial genera with restoration. Following this, we observed a decreased relative abundance of three periodontal bacteria (*Capnocytophaga*, *Prevotella 7*, and *Leptotrichia*) and increased relative abundance of two commensal bacteria (*Streptococcus* and *Rothia*), consistent with a better 3-year disease-specific survival (a cutoff of Kaplan–Meier survival curve test *p* < 0.3 at 36 months) (**Figure 5B**). In particular, the postsurgery restoration of *Prevotella 7* was statistically significant in the surveyed patients (survival rate of 87% vs. 56% at 24 months, *p* = 0.0074; 84% vs. 56% at 36 months, *p* = 0.0065). Univariate regression analyses did not observe a significant association of clinical features with disease-specific survival, although patients without post-surgery chemotherapy, not smoking, no alcohol consumption, a lower T stage, or HPV infection might have better survival (**Supplementary Figure S3**).

## Discussion

Here, we evaluated the changes in the oral microbiome following the surgical management of HNSCC and its association with clinical outcomes. Our data provide insight into the oral microbiome dynamics during the management of HNSCC from surgery to recovery, and these changes are associated with patient outcomes. In particular, the restoration of the oral microbiome towards a normal healthy control is associated with improved 3-year disease-specific survival and the postsurgery radiotherapy treatment.

The oral microbiome diversity between pretreatment and healthy control samples on measures of α-diversity (richness, Shannon, and Simpson index) and β-diversity (GUniFrac and Bray–Curtis) is similar to other studies evaluating the oral microbiome in HNSCC (13, 14, 16). Interestingly, following the surgical management of HNSCC, the oral microbiome was found to have a reduced α-diversity and altered β-diversity when compared to preoperative treatment and healthy controls. Through the 6 months posttreatment, there was no significant change in the α-diversity or change in the clustering of β-diversity in the serial oral rinses, but they remained significantly different from pretreatment and healthy controls. These β-diversity changes are similar to those noted during radiotherapy for nasopharyngeal carcinoma, but not α-diversity, which may reflect differences in tumor locations and etiology (21, 22). These posttreatment changes are also similar to the changes in a cohort of predominantly HPV-positive oropharyngeal carcinoma. However, a longer-term follow-up at 2 years showed a trend towards restoration of baseline diversities and richness of the microbiome, most likely secondary to the time of follow-up oral rinse collections and different sample cohorts (23).

Comparisons between pre- and posttreatment oral rinse samples showed that several bacteria are discriminant between the two time points. Furthermore, certain bacteria genera had trends of altered abundance that were significantly different from baseline pretreatment sampling. *Streptococcus*, *Rothia*, and *Gemella* increased in abundance from pre- to postsurgery at 6 months; meanwhile, *Fusobacterium*, *Capnocytophage*, *Preveotella 7*, *Allopreveotella*, and *Leptotrichia* decreased. These trends indicate a directional posttreatment change towards bacterial communities that are similar to healthy controls. This also follows the reverse pattern of an increased relative abundance of *Fusobacterium* from healthy controls to patients with premalignant oral cavity lesions and finally patients with oral cavity squamous cell carcinoma that has been previously noted (14). These data combined suggest that there is a niche set of bacterial communities that partially return to healthy posttreatment levels.

Interestingly, *Neiserria*, *Porphyromonas*, and *Haemophilus* trended significantly lower than healthy controls and preoperative relative abundances, while *Lactobacillus* had the opposite trend to increased relative abundances postoperatively compared to healthy controls and pretreatment. *Neiserria*, *Porphyromonas*, and *Haemophilus* in the oral cavity have been shown to be important in the entero-salivary nitrate–nitrite–nitric oxide pathway by acting as potent nitrate-reducing bacteria (24). Conversely, *Lactobacillus* has been reported as a poor nitrate-reducing bacteria. Overall, these may lead to the loss of homeostasis of nitric oxide and affect processes including angiogenesis, apoptosis, and immune responses (25). Another reason for this may be secondary to the change in saliva production in this cohort, as a large number of patients underwent postoperative radiotherapy. This change in saliva production lowers the ability to buffer pH changes, influence the microbial ecosystem, and permit and increase the number of acidogenic bacteria such as *Lactobacillus* (26).

Evaluating patient outcomes in relation to the relative abundances of bacterial communities over time demonstrated that factors including radiotherapy treatment and 3-year disease-specific survival were significantly associated with changes in a certain genus of bacteria. The five genera of bacteria including *Capnocytophagia*, *Prevotella 7*, *Leptotrichia*, *Streptococcus*, and *Rothia* were noted to be associated with DSS of patients. In particular, *Prevotella 7* was significantly associated with DSS, with patients showing a decrease in abundance of *Prevotella 7* posttreatment having a significantly better DSS. Given that the oral microbiome plays a vital role in local and systemic immune homeostasis, these changes may indicate a restoration of the niche microbiome communities towards that of healthy persons that then participate in maintaining a healthy environment. Alternatively, these dynamic changes may be bystanders that are influenced by the presence or absence of tumors and their associated changes. These also suggest that manipulation of the oral microbiome may be a potential treatment option to improve patient outcomes in HNSCC, analogous to the use of *Lactobacillus brevis* in periodontitis and mucositis (27, 28).

A few microbial species can directly cause cancer, such as *Helicobacter pylori* and *Fusobacterium nucleatum*, and their roles in gastric cancer and colorectal cancer, respectively (29–31). However, the majority of cancer-associated bacteria have long been considered to be opportunistic; the colonization of some “complicit” bacteria may promote carcinogenesis through immunomodulation but are insufficient to cause cancer (32, 33). A knowledge gap still separates the complex links of the evolutionary dynamics between the host immune system, the tumorigenic processes, and the commensal microbiome and microbial functions. Because both host and microbiota genetic heterogeneities are associated with carcinogenesis (34), clinical observation targeted at microbiota may provide an opportunity to intervene in cancer diagnostics and treatment that we are just beginning to uncover.

A limitation of this study was the inclusion of relatively conservative postsurgery time points. Additional (shorter and longer) time periods may reveal different trends. However, this cohort benefits from the complete oral rinse sample collection at these time points across the study in all patients. Another limitation is that there was no longitudinal collection of healthy controls to evaluate the changes in healthy persons across time that may have some bearing on the changes across time in HNSCC patients posttreatment. Finally, we were unable to fully evaluate the impact of diet, lifestyle, or medication use, and its impact on the serial oral microbiota findings, which warrant further validation using additional cohorts to evaluate the potential of oral microbiota as a biomarker for clinical diagnostics and interventions in HNSCC.

## Conclusions

In summary, we have shown that the oral microbiome dysbiosis associated with HNSCC is dynamic, and, following treatment, there is a trend towards the restoration of niche communities of the microbiome that approximate healthy persons. These dynamics of the oral microbiome posttreatment are also associated with patient outcomes and may serve as potential biomarkers for patient management in HNSCC.

## Data Availability Statement

The datasets presented in this study can be found in online repositories. The names of the repository/repositories and accession number(s) can be found below: PRJNA744870, https://www.ncbi.nlm.nih.gov/bioproject/PRJNA744870.

## Ethics Statement

The studies involving human participants were reviewed and approved by The Joint Chinese University of Hong Kong—New Territories East Cluster Clinical Research Ethics Committee. The patients/participants provided their written informed consent to participate in this study.

## Author Contributions

Study design: ZC, JC, and PC. Support with materials: CN, KC, SF, LL, EL, ZY, EW, and J-WL. Experiments: PW, SF, and PL. Data interpretation and analysis: ZC, JC, PC, LC, PL, QM, and KM. Manuscript writing: all authors. All authors contributed to the article and approved the submitted version.

## Funding

This work was partially supported by funding agents from the Stanley Ho Medical Foundation (JC), the Research Grants Council of the Hong Kong Special Administrative Region, China (project numbers CUHK 14108818, JC; and project number CUHK 14161017—ZC), the Direct grant (project number CUHK 4054350—ZC), and the Seed Fund for Gut Microbiota Research (PC) from the Faculty of Medicine, The Chinese University of Hong Kong, Hong Kong Special Administrative Region, China. The funders had no role in the study design, data collection, analysis, interpretation, or writing of the report.

## Conflict of Interest

The authors declare that the research was conducted in the absence of any commercial or financial relationships that could be construed as a potential conflict of interest.

## Publisher’s Note

All claims expressed in this article are solely those of the authors and do not necessarily represent those of their affiliated organizations, or those of the publisher, the editors and the reviewers. Any product that may be evaluated in this article, or claim that may be made by its manufacturer, is not guaranteed or endorsed by the publisher.

## Supplementary Material

Supplementary Figure 1 The composition of oral microbiota summarized at the phylum level between healthy controls (Ctrl) and HNSCC patients before surgery (Pre) and after surgery at 1 (M1), 3 (M3), and 6 months (M6). The percentage of each bacterial phylum was plotted in the bar chart, with mean abundance ± standard error of the mean in the table.

Supplementary Figure 2 The composition of oral microbiota summarized at the genus level between healthy controls (Ctrl) and HNSCC patients before surgery (Pre) and after surgery at 1 (M1), 3 (M3), and 6 months (M6). The percentage of each bacterial genus was plotted in the bar chart, with mean abundance ± standard error of the mean in the table. Bacterial genera with ≥1% relative abundance in at least one sample were retained.

Supplementary Figure 3 Univariate regression analysis of clinical features associated with disease-specific survival.
