# Supplementary figures and images for: Restoration of the Oral Microbiota After Surgery for Head and Neck Squamous Cell Carcinoma Is Associated With Patient Outcomes
Source: Front Oncol. 2021 Oct 6;11:737843. doi: 10.3389/fonc.2021.737843 (PMC8527003; doi:10.3389/fonc.2021.737843)

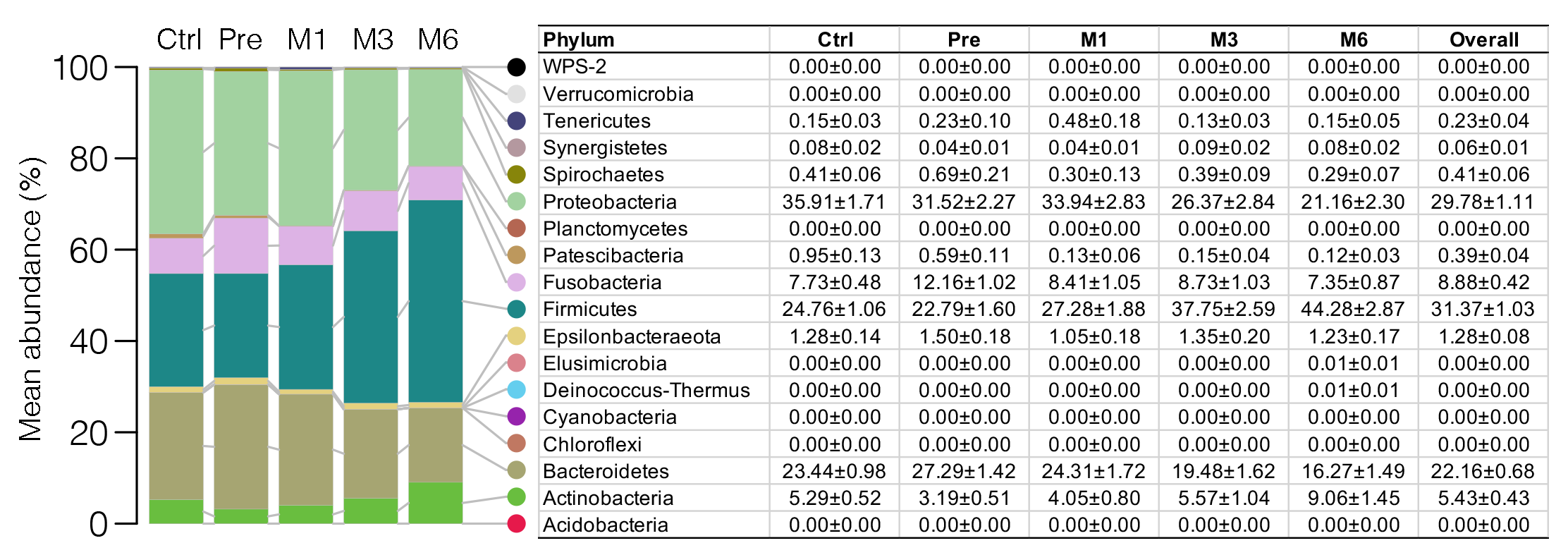

Supplement: Supplementary Figure 1 — The composition of oral microbiota summarized at the phylum level between healthy controls (Ctrl) and HNSCC patients before surgery (Pre) and after surgery at 1 (M1), 3 (M3), and 6 months (M6). The percentage of each bacterial phylum was plotted in the bar chart, with mean abundance ± standard error of the mean in the table. [file Image_1.tif]

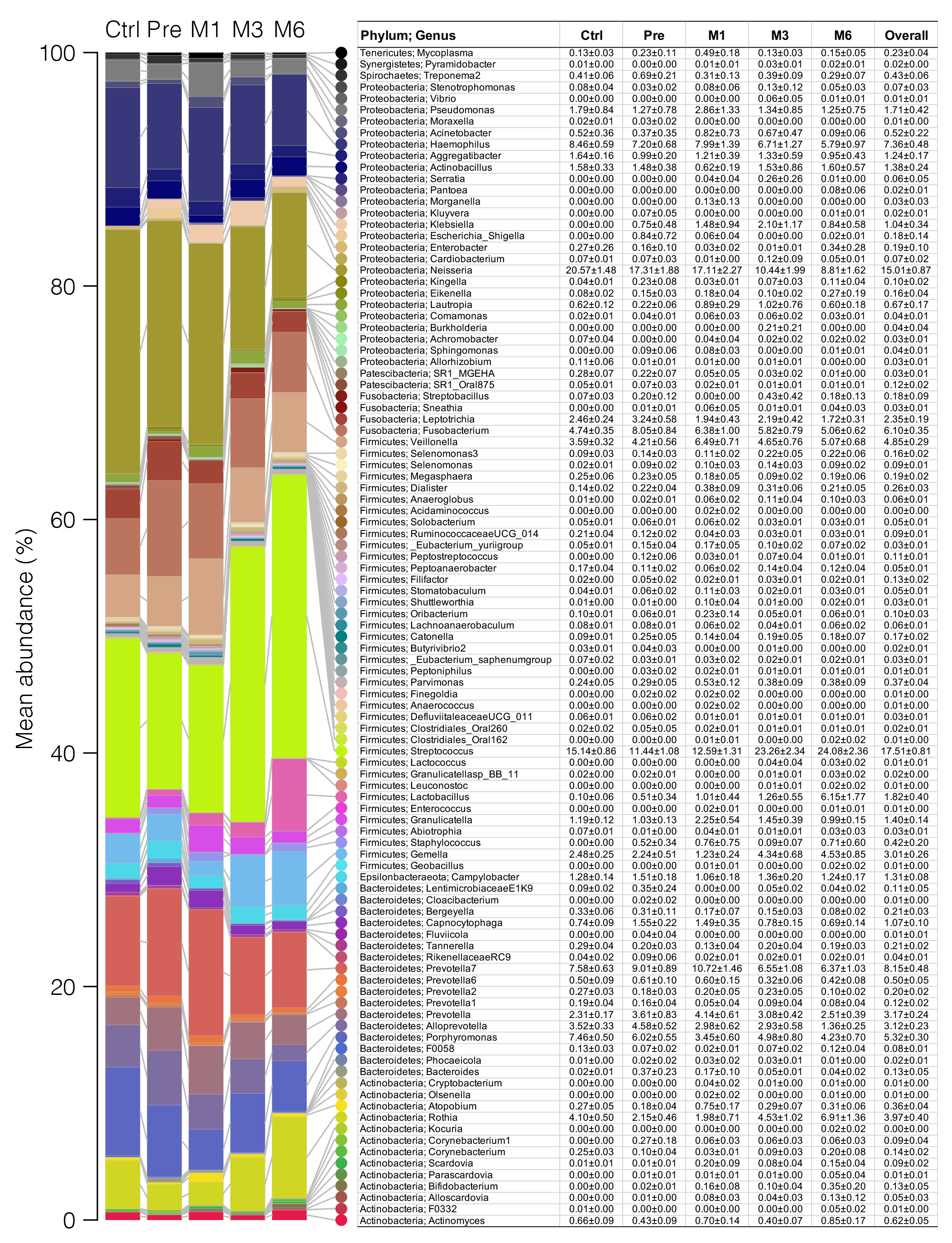

Supplement: Supplementary Figure 2 — The composition of oral microbiota summarized at the genus level between healthy controls (Ctrl) and HNSCC patients before surgery (Pre) and after surgery at 1 (M1), 3 (M3), and 6 months (M6). The percentage of each bacterial genus was plotted in the bar chart, with mean abundance ± standard error of the mean in the table. Bacterial genera with ≥1% relative abundance in at least one sample were retained. [file Image_2.tif]

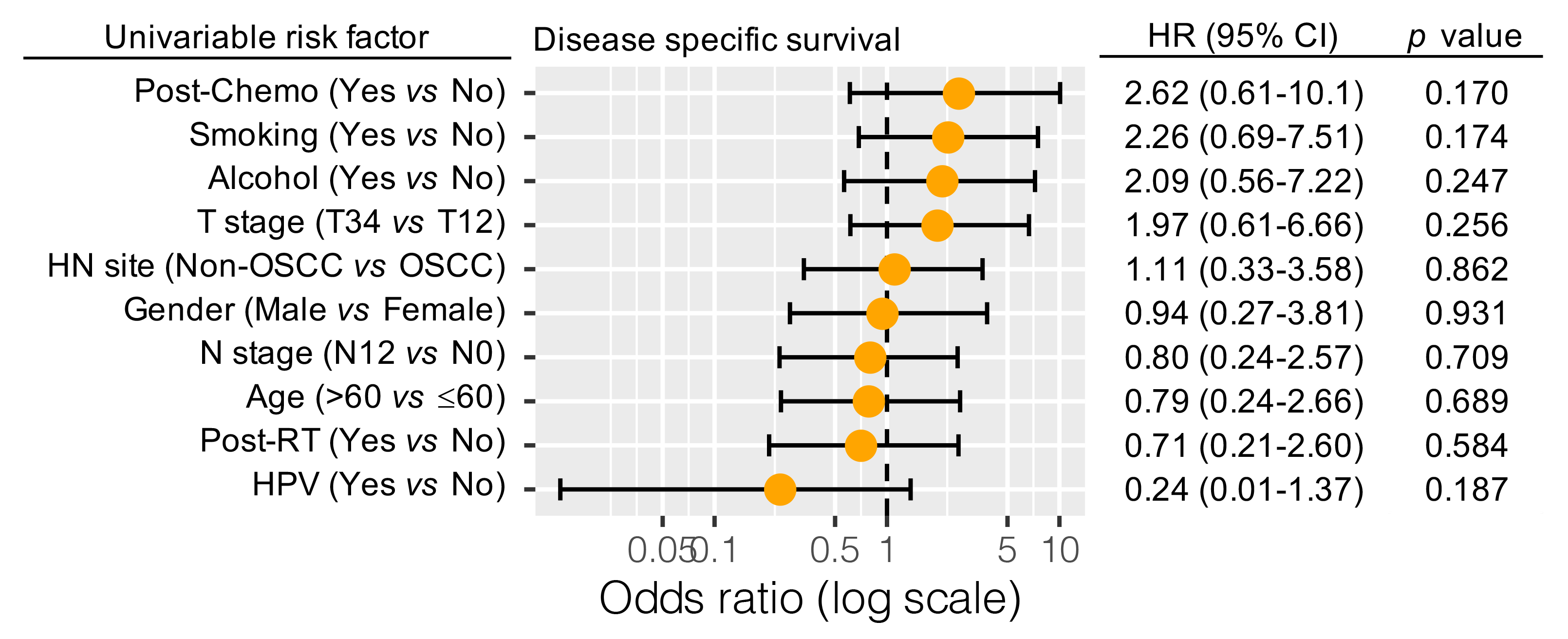

Supplement: Supplementary Figure 3 — Univariate regression analysis of clinical features associated with disease-specific survival. [file Image_3.tif]
